# Performance and robustness of small molecule retention time prediction with molecular graph neural networks in industrial drug discovery campaigns

**DOI:** 10.1038/s41598-024-59620-4

**Published:** 2024-04-16

**Authors:** Daniel Vik, David Pii, Chirag Mudaliar, Mads Nørregaard-Madsen, Aleksejs Kontijevskis

**Affiliations:** Amgen Research Copenhagen, Amgen Inc., 2100 Copenhagen, Denmark

**Keywords:** Chromatography, Machine-learning, Retention time, Small molecule, Applied artificial intelligence, Pharmaceuticals, Cheminformatics, Mass spectrometry

## Abstract

This study explores how machine-learning can be used to predict chromatographic retention times (RT) for the analysis of small molecules, with the objective of identifying a machine-learning framework with the robustness required to support a chemical synthesis production platform. We used internally generated data from high-throughput parallel synthesis in context of pharmaceutical drug discovery projects. We tested machine-learning models from the following frameworks: XGBoost, ChemProp, and DeepChem, using a dataset of 7552 small molecules. Our findings show that two specific models, AttentiveFP and ChemProp, performed better than XGBoost and a regular neural network in predicting RT accurately. We also assessed how well these models performed over time and found that molecular graph neural networks consistently gave accurate predictions for new chemical series. In addition, when we applied ChemProp on the publicly available METLIN SMRT dataset, it performed impressively with an average error of 38.70 s. These results highlight the efficacy of molecular graph neural networks, especially ChemProp, in diverse RT prediction scenarios, thereby enhancing the efficiency of chromatographic analysis.

## Introduction

Chromatographic techniques play a pivotal role in chemical analysis, enabling the separation and identification of compounds within complex mixtures. One critical parameter in chromatography is the retention time (RT). Accurate prediction of small molecule RT can greatly expedite compound identification and data interpretation in applications such as metabolomics, chemical quality control, and beyond.

Parallel organic synthesis of small molecules has in recent years become an integral part of industry-scale drug discovery. Particularly, robust nanoscale high-throughput hit resynthesis supporting large-scale screening technologies such as DNA-encoded library screening. Such small-scale platforms inherently rely on starting material in small quantities (typically nmol range), thus yielding nanoscale amounts of compound of interest. To obtain discrete compounds for biological assaying, these are isolated through preparative reverse-phase ultra-high performance liquid chromatography. Improved purification outcomes can be attempted by pre-purification analysis of compounds through the concept of gradient scouting runs. During scout runs, crude material is sacrificed in order to identify optimal gradient conditions for compound separation. However, in a small-scale parallel chemistry setting – producing minute amounts of synthetic small molecules in the order of thousands – the time and material constraints disfavor such gradient scouting runs. Accurate predictions of small molecule RT would enable the use of in silico analytical scouting runs to select optimal compound-specific purification methods and gradients. Such achievement has great implications on preparative liquid chromatography efforts, benefitting especially: (1) Conserving product material, as physical chromatography scout runs can be omitted; (2) Shorter, focused separation runs thereby reducing instrument cycle-time, and solvent consumption; (3) Better separation of isomeric compounds (e.g., diastereomers and regioisomers).

We here report the examination of machine-learning (ML) models to predict RT, aiming to enhance the efficiency and accuracy of chromatographic analysis, specifically in the context of a high-throughput parallel synthesis workflow employed in industrial-scale drug discovery projects. Importantly, we are not providing a comprehensive review or exhaustive benchmark of recent ML methods. Rather, we are examining the application of several popular modelling frameworks in an industry setting, where reliability and robustness of the models are all-important – both in terms of predictive performance, as well as model framework, and associated codebase. As the number of reports on innovative and unique model architectures increases (e.g. transformer based models)^1,2^ our focus remains on investigating the time-dependent robustness of models within three firmly established frameworks: XGBoost^3^, ChemProp^4–6^, and DeepChem^7^. We examine these frameworks in the context of the public METLIN SMRT dataset^8^, as well as a proprietary dataset of 7552 small molecule compounds from our parallel synthesis platform. Critically, the proprietary dataset enables the unique examination of model performance decay over time, closely mirroring the changing chemistries of industrial drug discovery campaigns.

## Results

**Figure 1:**
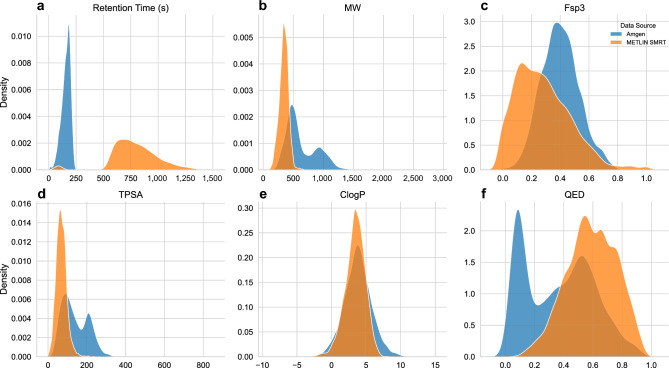
Kernel density estimates visualizing the distribution of observations in the two datasets: proprietary Amgen dataset and the public METLIN SMRT dataset. Retention times and five calculated descriptors are shown to exemplify the dissimilarity between the two datasets.

Our examination is based on 7552 compounds which represent a diverse set of chemical series accumulated over the course of several years and drug discovery campaigns. This dataset has distinct properties compared to the public benchmark dataset, METLIN SMRT (Fig. 1), which is a milestone in RT prediction that has fueled improvements in solving the RT prediction task^8–13^.

While the importance of large public datasets cannot be understated, it is important to recognize that such datasets have implicit biases and limitations which can lead to poor transferability when models are later trained on non-standard datasets^14^.

We trained a series of different models in combination with three sets of descriptors: extended connectivity fingerprints (ECFP), which is a set of binary substructure-based features representing the absence and presence of distinct chemical substructures in a molecule; a set of 200 RDKit descriptors (i.e. a wide range of calculated physicochemical properties) from the DeepChem python library, as well as a range of ChemAxon LogD at different pH values. Calculated LogD has been shown earlier to correlate well with RT^15,16^. Four model types were examined: (1) XGBoost^3^, gradient boosted trees; (2) AttentiveFP, a molecular graph neural network with an attention-mechanism^17^; (3) a fully connected neural network (FCNN); and (4) ChemProp, a molecular graph neural network based on *directed* message-passing^4^. XGBoost and ChemProp were each combined with the three descriptor sets (ECFP4, RDKit descriptors, and LogDs). AttentiveFP relies solely on the molecular graph representation only and is not able to take advantage of additional descriptors. In addition, we included a FCNN as it was applied to the METLIN SMRT dataset in the original report by Domingo-Almenara et al.^8^ Model evaluation was based on fivefold cross validation with hyperparameter optimization is reported in Tables 1 and 2. The molecular graph neural network models (AttentiveFP and ChemProp) outperformed XGBoost and the FCNN. The best performing model based on validation schema was ChemProp combined with RDKit descriptors.

**Table 1:** General model performance.

	MAE			RMSE			R^2^		
	Mean	Median	sd	Mean	Median	sd	Mean	Median	sd
ChemProp_RDKit	6.05	6.13	0.23	9.46	9.49	0.45	0.95	0.95	0.01
ChemProp_LogD	6.48	6.48	0.27	10.87	10.78	0.49	0.93	0.93	0.01
ChemProp_ECFP4	7.16	7.13	0.27	10.6	10.64	0.26	0.93	0.93	0.00
AttentiveFP	9.84	9.56	1.34	13.19	12.9	1.55	0.89	0.9	0.02
FCNN_ECFP4	11.24	11.37	0.5	16.57	16.53	0.67	0.84	0.84	0.01
XGBoost_RDKit	12.27	12.22	0.32	17.35	17.21	0.35	0.82	0.82	0.01
FCNN_RDKit	13.61	13.66	0.14	18.16	18.1	0.09	0.80	0.80	0.00
XGBoost_ECFP4	13.68	13.59	0.14	19.84	19.9	0.18	0.76	0.76	0.00
XGBoost_LogD	15.19	15.21	0.22	20.34	20.3	0.28	0.75	0.75	0.01
FCNN_LogD	16.57	16.26	0.66	21.67	21.22	0.85	0.72	0.73	0.02

Models trained on Amgen data. Mean, median and standard deviation (sd) are based on fivefold cross validation. Scores are Mean Absolute Error (MAE) in seconds, Root Mean Square Error (RMSE) in seconds and R^2^.

**Table 2:** Statistical Post-Hoc test, multiple comparisons of RT prediction models.

	AttentiveFP	ChemProp_ECFP4	ChemProp_LogD	ChemProp_RDKit	FCNN_ECFP4	FCNN_LogD	FCNN_RDKit	XGBoost_ECFP4	XGBoost_LogD
AttentiveFP									
ChemProp_ECFP4	0.018								
ChemProp_LogD	< 0.001	0.081							
ChemProp_RDKit	< 0.001	< 0.001	0.628						
FCNN_ECFP4	1	< 0.001	< 0.001	< 0.001					
FCNN_LogD	< 0.001	< 0.001	< 0.001	< 0.001	< 0.001				
FCNN_RDKit	< 0.001	< 0.001	< 0.001	< 0.001	< 0.001	< 0.001			
XGBoost_ECFP4	< 0.001	< 0.001	< 0.001	< 0.001	< 0.001	< 0.001	1		
XGBoost_LogD	< 0.001	< 0.001	< 0.001	< 0.001	< 0.001	0.116	< 0.001	0.003	
XGBoost_RDKit	< 0.001	< 0.001	< 0.001	< 0.001	0.057	< 0.001	0.002	< 0.001	< 0.001

Bonferroni corrected *p*-values from Conover’s test for pairwise dissimilarity between models based on Mean Absolute Error (MAE) scores from each cross-validation fold (n = 5). Prior to the post-hoc test a non-parametric Friedmans test was performed across all models (uncorrected *p*-value: 0.000014).

**Figure 2:**
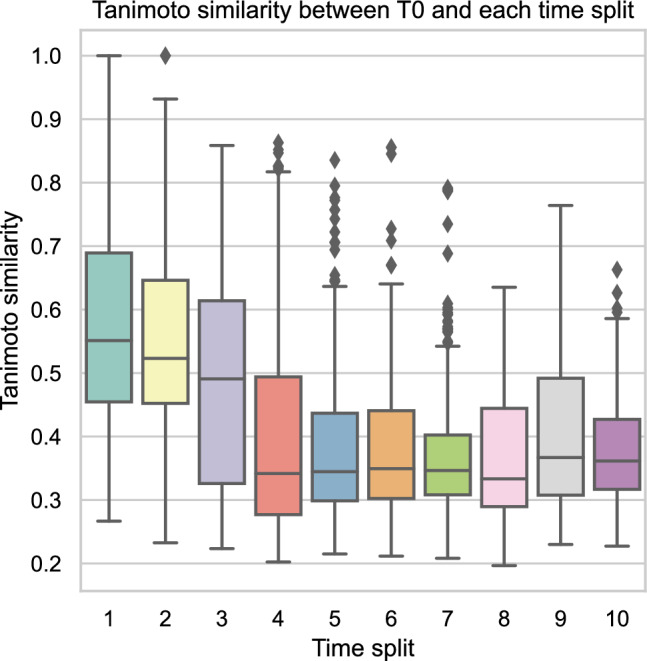
Tanimoto similarity of the nearest neighbor from the T0 training dataset to each compound of each time split (T1–T10). Tanimoto similarity calculated based on ECFP4-1024 fingerprints.

**Figure 3:**
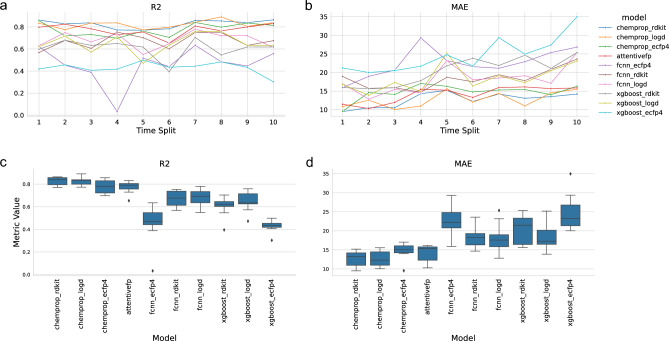
Performance of RT models trained on the T0 time split and evaluated on ten chronologically derived time splits (T1–T10). Panel (**a**–**b**) show the model performance at each timepoint with the time splits on x-axis and R^2^ and MAE (seconds) shown on y-axis. Panel (**c**–**d**) boxplots comparing models aggregated across all timepoints. Models shown on x-axis, and with R^2^ and MAE (seconds) on y-axis in panel (**c**) and (**d**), respectively.

Individual drug discovery campaigns typically navigate distinct chemical spaces, exploring chemical series based on hit-matter identified in various ways (e.g., DNA-encoded library screening). This can be a challenge for ML models as the historical data on which they are trained may differ substantially from the chemical space under current investigation. A model will have to generalize well to such uncharted chemical space to be practically useful for a new drug discovery campaign. To address this question regarding time-dependent performance decay, we next sought to examine model robustness by training models on data that had been split temporally (rather than by scaffold-splitting). To do this, we designed a new training regime for the models, where the data was split according to the time of acquisition. Data was sorted according to acquisition date and split in half, the earliest half (T0) was used for model training, while the latter half was split again in ten equal bundles (T1–T10) representing temporal shifts in the chemistry of interest – with decreasing chemical similarity from the training data (Fig. 2). This training regime closely mirrors the changing priorities and interests of ongoing drug discovery projects where new targets and new chemical series with different properties come into focus. Again, the molecular graph-based models (ChemProp and AttentiveFP) outperformed XGBoost and the FCNN (Fig. 3). In particular, ChemProp in combination with RDKit descriptors appear to be very robust over time (Fig. 3a,b). Thus, a RT model based on ChemProp with RDKit descriptors emerges as accurate and robust for solving RT prediction tasks.

**Figure 4:**
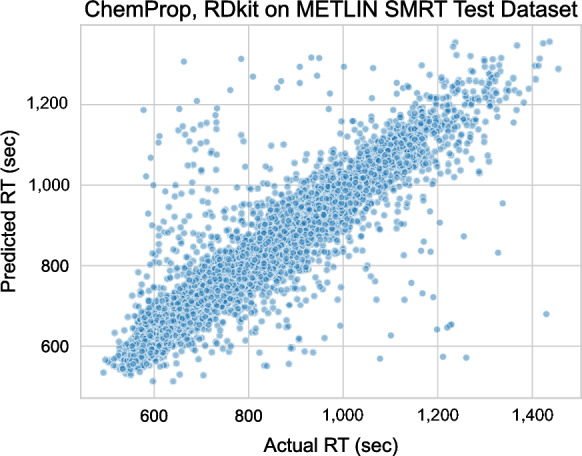
ChemProp model with RDKit descriptors trained on the METLIN SMRT dataset. Scatterplot showing predicted RT (seconds) vs actual RT (seconds) for the retained compounds in the test split.

Next, we decided to explore its applicability in a wider context by predicting RT for METLIN SMRT dataset. The METLIN SMRT dataset is notably different from our dataset, both in terms of chemical diversity and in terms of chromatographic system (Fig. 1). Figure 4 demonstrates the relationship between actual and predicted RT for the ChemProp model (with RDKit descriptors) trained on the METLIN SMRT dataset. The ChemProp model could accurately predict RT with mean absolute error (MAE) of 38.7 s, with RMSE 67.50 s, and R^2^ = 0.84, which is on par with the recently reported MAE scores of 34–39 s^9–13^. It is important to note that our evaluation was based only on the chromatographically retained compounds of the METLIN SMRT dataset.

## Discussion

Several current studies have explored diverse ML models for RT prediction, showcasing the field's dynamic evolution^8–13^. Notably, Osipenko et al.^18^ reported the application of a message-passing neural network architecture to the RT prediction task and achieved comparable results on the METLIN SMRT dataset as well as a range of other public datasets. However, their approach differs significantly from ours, as ChemProp uses *directed* message-passing (i.e. explicitly considering the directionality of edges in molecular graphs during the message passing process, capturing the order and orientation of chemical bonds) which has been shown to positively affect performance^4^. In addition, rather than applying simple random data splitting, we apply scaffold splitting. Scaffold-splitting is known to seemingly decrease the performance of the model; however, it generally leads to better generalizability of the model and reduces the risk of overfitting^4^.

Recently, impressive results have been achieved by Kang et al.^19^ who constructed a graph convolutional neural network which in addition to an alternative message-passing procedure has a depth of no less than 16 layers. Both Osipenko et al.^18^ and Kang et al.^19^ do not apply additional features to their models but rely solely on the graph representations. This is similar to the AttentiveFP model examined in this report^17^, which only relies on the molecular graph representation, rather than incorporating additional features such as ECFP4 fingerprints or physicochemical descriptors. In our report we show, however, that physicochemical descriptors (i.e. RDKit descriptors or calculated LogD descriptors) in combination with graph convolutional neural networks can provide accurate prediction results, as well as time-dependent robustness and generalizability.

The apparent success of graph-based methods with the RT prediction task likely reflects that graphs are effective representations of the 2D structure of molecules (as the graph structure enables effective capture and propagation of complex relationships and dependencies)^4,6,20^. This might also explain the positive effect of RDKit features (compared to ECFP4 features) as they include a range of calculated physicochemical properties (such as total polar surface area and fraction of sp^3^-hybridization)^21^ which likely relate more directly with chromatographic retention compared to isolated substructures. RT prediction is a task where it can be assumed that similar molecules will have similar RT. This is in direct contrast with other molecular property prediction tasks which sometimes suffer from so-called *activity-cliffs*. Interestingly, Dablander et al.^22^ recently reported on modelling such activity-cliffs, and found that in certain cases substructure-based fingerprints (i.e. ECFP4 fingerprints) outperform both physicochemical descriptors (i.e. RDKit features) and graph convolutional neural networks, reflecting that some tasks are more accurately modelled by the absence or presence of individual substructures – rather than global molecular properties. This underlines the importance of testing different types of molecular features when modelling different molecular properties.

In summary, this study explored ML models for predicting RT in chromatographic analysis, with a focus on high-throughput drug discovery. ChemProp, a molecular graph neural network, emerged as a robust choice for accurate RT prediction, both in our specialized dataset and the benchmark METLIN SMRT dataset. The study highlighted the adaptability of ChemProp to different chemical contexts, showing its efficacy in our proprietary parallel synthesis dataset and demonstrated its value in navigating evolving chemical spaces over time. Overall, our research underscores the potential of molecular graph neural networks in enhancing RT prediction accuracy and efficiency for diverse chemical analyses, propelling advancements in cheminformatics and compound identification.

## Methods

### RT acquisition by liquid chromatography mass spectrometry

Small molecules were analyzed by ultra-high performance liquid chromatography on an Agilent 1290 Infinity II LC System coupled to a time-of-flight mass spectrometer Agilent 6230B with a dual electrospray ion source and a Diode Array Detector (Agilent Technologies, Santa Clara, CA) using an ACQUITY Premier UPLC BEH C18 column (1.7 µm, 2.1 × 50 mm, Waters Corporation, Milford, MA). We used a mobile phase of solvent A (0.1% (v/v) formic acid in Milli-Q water) and solvent B (0.1% (v/v) formic acid in methanol) with a gradient consisting of 5% B for 0.2 min, 5 to 100% B in 3.8 min, 100% B for 0.5 min, 100 to 5% B in 0.1 min, and 5% B for 0.9 min with a constant flow rate of 0.75 mL/min. Raw data was processed using Agilent MassHunter Qualitative Analysis (v. B.07.00). RT was defined as the centered peak apex of the target compound measured from the start of injection and verified through manual inspection.

### Data preparation and splitting

Molecules containing stereocenters and common tautomeric motifs were preprocessed prior to model training. Stereoisomers with RT differences exceeding 10 s were removed, otherwise RT of stereoisomers was averaged, and a racemic mixture was used as a new data point. This yielded 7552 RT datapoints. A 10% scaffold split holdout served as the test set, while the remaining 90% was split further into validation/train dataset pairs using scaffold splitting for fivefold cross validation.

Time-dependent performance decay was analyzed using 20 equidistant splits based on date of compound synthesis. Splits 1–10 were merged into a training (T0) set, the rest remained as chronological test datasets (T1–T10). Tanimoto similarity between the sets was calculated using ECFP4 1024-bit fingerprints. Each compound from a T1–T10 bundle was compared to all compounds in T0 set to find its closest nearest neighbor with the highest Tanimoto similarity.

### Molecular descriptors and representations

For model input we explored a series of molecular descriptors and representations:

- ECFP4 fingerprints (2048 bits, radius 2), DeepChem ‘CircularFingerprint’ featurizer.
- Normalized RDKit descriptors (200 descriptors, excluding BCUT2D), DeepChem ‘RDKitDescriptors’ featurizer.
- LogD values calculated at 16 pH levels (0.0–7.4 with 0.5 pH bins), ChemAxon cxcalc module.
- Molecular graph convolutions, DeepChem ‘MolGraphConvFeaturizer’.
- Directed-Message Passing Neural Network embeddings, ChemProp (default settings.)

### Model training

Models were trained on a cluster with 20 CPUs, GPU (1 × Nvidia V100) and 128 GB RAM. Four model types were trained, each optimized with fivefold cross validation:

- AttentiveFP: Hyperparameter optimization for 100 epochs over 20 iterations using DeepChem implementation (v.2.7.1). Parameters included layers (1–6), graph feature size (30–300), dropout rate (0–0.5), learning rate (0.0001–0.01), and weight decay penalty (0.00001–0.01), optimized via Hyperopt and TPE algorithm^23,24^.
- ChemProp: Hyperoptimization for 100 epochs over 20 iterations with default settings from ChemProp implementation (v.1.60).
- XGBoost: 20 iterations of 100 estimators using Hyperopt and TPE algorithm. Parameters searched: learning rate (0.01–0.3), max depth (3–10), subsample (0.7–1.0), gamma (0–1), column sample by tree (0.7–1.0), minimum child weight (1–10), and regularization coefficients alpha (1e−10–1.0) and lambda (1e−10–1.0), with early stopping.
- Fully connected Neural Network: Hyperparameter optimization for 100 epochs over 20 iterations using DeepChem implementation (MultitaskRegressor). Optimized parameters were dropout rate (0–0.5), learning rate (0.0001–0.01), and weight decay penalty (0.00001–0.01). Layers were fixed (1000, 500, 200, 100), activation function was set as ReLU, weight decay penalty type was set as L2.

For statistical evaluation of model performance, a non-parametric Friedmans test was applied followed by Conover’s test for post-hoc analysis with Bonferroni correction^25,26^.

### Training ChemProp on METLIN SMRT data

The chemical structures from METLIN SMRT data were converted from InChI to SMILES strings using RDKit Chem module. Next, compounds with RT below 200 s were excluded as ‘non-retained’ compounds. This resulted in 77,901 RT datapoints for training. Model training (i.e., hyperparameter optimization and retraining) was performed in the same way as described above for ChemProp model training, however, fivefold cross validation was not performed.

## Data Availability

The METLIN SMRT data is available through the supporting information of Domingo-Almenara et al.^8^ and can be found here: https://figshare.com/ndownloader/files/18130628. While we cannot openly share our proprietary dataset used in this publication due to intellectual property concerns, we are open to discussing partial disclosure of the dataset on an individual case-by-case basis. Contact the corresponding author with enquiries. Code for reproducing the results is available here: https://github.com/danielvik/arc_rtpred

## Abbreviations

LC: Liquid chromatography
RT: Retention time
MAE: Mean absolute error
RMSE: Root mean square error
sd: Standard deviation
TPE: Tree-Parzen estimator
ECFP4: Extended connectivity fingerprints, radius 2
FCNN: Fully connected neural network
